# Multi-Objective Optimization of a Multi-Cavity, Significant Wall Thickness Difference Extrusion Profile Mold Design for New Energy Vehicles

**DOI:** 10.3390/ma17092126

**Published:** 2024-04-30

**Authors:** Xuda Xu, Feng Jiang, Jianxiang Li, Hongfeng Huang, Chunli Jiang

**Affiliations:** 1 School of Material Science and Engineering, Central South University, Changsha 410083, China; 2 Guangdong Hoshion Aluminium Co., Ltd., Zhongshan 528463, China; 3 Key Laboratory of New Processing Technology for Nonferrous Metal & Materials, Guilin University of Technology, Ministry of Education, Guilin 541004, China; 4 Guangdong Institute of Special Equipment Inspection and Research Zhongshan Branch, Zhongshan 528400, China

**Keywords:** Al-Mg-Si alloy, extrusion, die design, response surface method, NSGA2

## Abstract

With the rapid development of the new energy vehicle market, the demand for extruded profiles for battery trays, mainly characterized by significant wall thickness differences in multiple chambers, is increasing, posing new challenges to production and quality control. This study examines the multi-objective optimization problem in the design process of aluminum profile dies with multi-cavity profiles and significant wall thickness differences. Using QFORM-extrusion professional aluminum extrusion finite element analysis software and the response surface analysis method, the standard deviation of the velocity (SDV), standard deviation of the pressure (SDP), and thick wall hydrostatic pressure (TWHP) on the profile section at the die exit are optimized. By analyzing the functional relationship between the key die structure parameters (the height of the baffle plates, the length of the bearing, and the height of the false mandrel) and the optimization objective, the optimal combination scheme of die structure parameters was obtained using the NSGA2 (non-dominated sorting genetic algorithm-2) multi-objective genetic optimization algorithm. The results show that, compared with the initial design scheme, the standard deviation of profile section velocity was reduced by 5.33%, the standard deviation of pressure was reduced by 11.16%, and the thick wall hydrostatic pressure was increased by 26.47%. The die designed and manufactured using this scheme successfully completed the hot extrusion production task, and the profile quality met the predetermined requirements, thus verifying the effectiveness of this study in optimizing the design of a multi-cavity aluminum profile die with significant differences in wall thickness for complex structures.

## 1. Introduction

Extrusion dies play a crucial role in aluminum profile production, and the optimization of die design is a key strategy for improving the service life of the dies and eliminating the abnormal production quality of the profile. In this critical technology field, scholars and industry experts have achieved significant success through in-depth theoretical research and rich practical experience. With the continuous advancement of computational technology, the application of finite element analysis (FEA) technology in extrusion process analysis and die design optimization has become increasingly widespread, promoting the development of related research and practice [1,2,3]. Professional finite element analysis software such as Deform-3D, HyperXtrude, and Qform Excitation not only improves the accuracy of die design but also significantly shortens the design cycle. This software, through its high-precision simulation capabilities, can predict material flow, stress distribution, temperature changes, and potential defects before actual manufacturing, effectively reducing the amount of trial and error, lowering production costs, and improving the quality and consistency of the final product [4,5,6,7,8,9,10].

**Figure 1:**
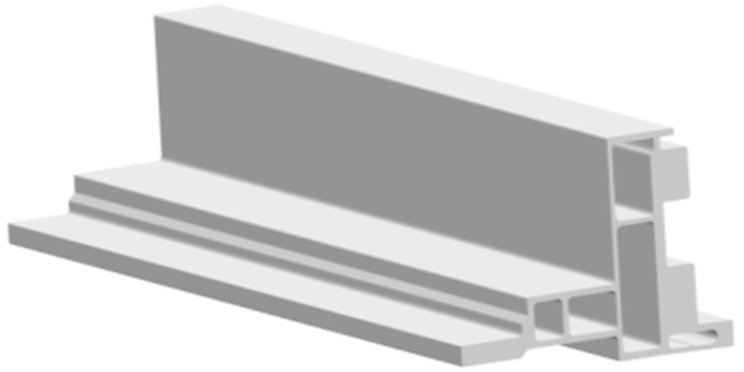
3D structural diagram of profiles.

**Figure 2:**
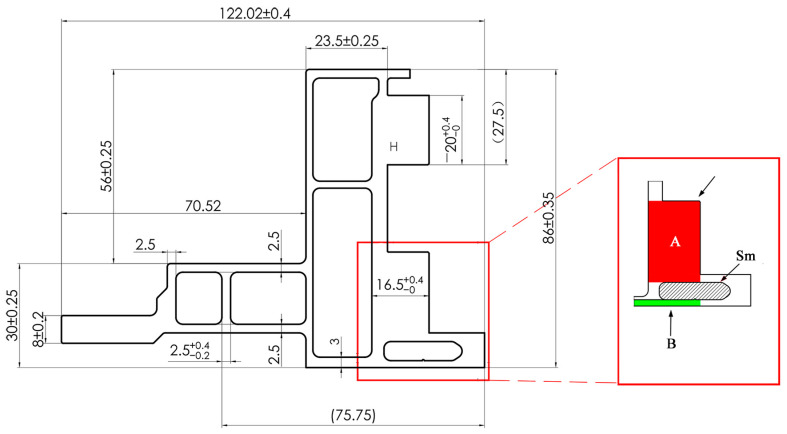
Dimensional tolerances and key structures of profiles (A: The area of thick wall, B: The area of thin wall, Sm: small mandrel).

**Figure 3:**
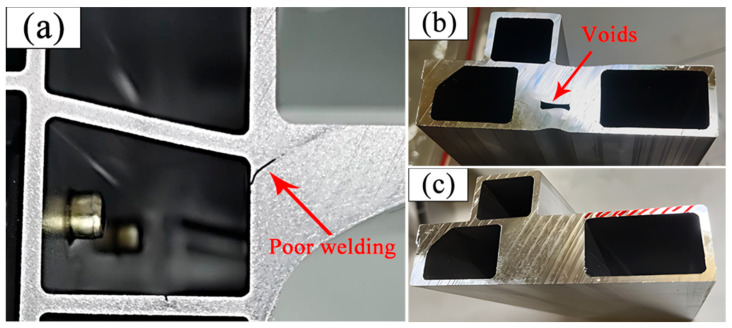
Dimensions and key structures of profile sections: (**a**) abnormal welding of profiles and holes caused by abnormal filling; (**b**) abnormal hole filling at the thick wall of profile 2; (**c**) normal cross-section of profile 2.

However, currently, most researchers explore indicators related to the quality of profile forming, usually using the standard deviation of cross-sectional flow velocity (SDV) as the main evaluation indicator, and based on this indicator, carry out corresponding die design optimization work [4,5,6,7,8,11,12]. The smaller the SDV value, the more uniform the distribution of the discharge velocity at various positions on the profile section, which is crucial for ensuring the dimensional accuracy and surface quality of the profile. This study suggests that this evaluation method is suitable for profiles with simple structures and a uniform wall thickness. However, with the rapid changes in the market, especially the widespread application of profiles in new energy vehicle structural components, there have been some changes in the cross-sectional structure of profiles. As shown in Figure 1 and Figure 2, the side beam profile in the battery tray assembly of new energy vehicles has the characteristics of multiple cavities, significant wall thickness deviation, and local mass concentration. The wall thickness deviation at different positions is close to tenfold, and in local key positions where the cross-sectional quality of the profile is concentrated, poor welding or even voids may occur, as shown in Figure 3. Due to the fact that such quality hazards are related to the safety of component use, the consequences, once they occur, are unimaginable. Therefore, when manufacturing this type of profile, we cannot only be satisfied with SDV optimization but also need to take filling and welding performance as the primary evaluation indicators for die design and optimization. This is to ensure that the product meets market demand and ensures safety.

Another problem brought about by the significant wall thickness difference is that there is a significant difference in hydrostatic pressure on both sides of the mandrel. When the stiffness of the mandrel is insufficient (affected by the shape and size), the mandrel will shift toward the low-pressure side (at the thick wall), potentially causing the profile size to exceed the standard or even irreversible plastic deformation of the mandrel, which has adverse effects on product quality and mold life. In addition, mandrel offset also leads to the formation of obstruction or flow promotion angles [13,14], making it more difficult to control the cross-sectional flow velocity of the profile and exacerbating the deterioration of SDV indicators. In summary, in response to the new challenges brought by the structural characteristics of multi-cavity profiles with significant wall thickness differences, selecting effective evaluation indicators and collaborating with SDV indicators for optimization are urgent problems that researchers need to solve.

The hydrostatic pressure inside the die welding chamber is an important factor affecting the welding performance of profiles and the filling ability of aluminum alloys [15,16,17]. The uniformity of the distribution of static water pressure on the cross-section can reflect the degree to which each core of a multi-cavity profile may experience deflection during the extrusion process due to pressure differences. Therefore, this article introduces new evaluation indicators, namely the static water pressure at thick walls (TWHP) and the standard deviation of pressure at each point of the profile cross-section (SDP), to characterize the welding performance, filling performance, and core stability at key positions. The above data can be obtained intuitively using finite element analysis software.

## 2. Research Objects and Methods

In this study, multi-objective optimization research was carried out for the extrusion die design of new energy vehicles with complex multi-cavity profiles and significant wall thickness differences. Based on QFORM 10.2.1 software, the Box–Behnken test was designed. Through response surface analysis, the functional relationships between three key design variables (the height of the baffle plates, the length of the bearing, and the height of the false mandrel) and three key product quality objectives (the standard deviation of the outlet velocity (SDV), the standard deviation of the pressure (SDP), and the thick wall hydrostatic pressure) were identified. At the same time, to improve the accuracy of the functional relationship, the flow stress constitutive equation of the alloy was considered and modified.

The Pareto optimal solution set was obtained by calculating the nonlinear function using the NSGA2 multi-objective genetic optimization algorithm. Combined with expert scoring and the TOPSIS method, the best scheme was selected from the Pareto solution set [18]. Finally, according to the optimization results, the die manufacturing was completed and successfully applied in production.

### 2.1. Constitutive Equation of 6061 Alloy with a Specific Composition during Hot Deformation

Utilizing a reasonable constitutive model is vital for accurately describing the deformation behavior of materials at high temperatures, under large strain, and with a high strain rate. At the same time, the composition differences between alloys lead to significant differences in flow deformation behavior even between alloys of the same series [19]. In order to ensure the accuracy of simulation, isothermal hot compression tests were carried out using a GLEEBLE-3500 thermal simulation testing machine for 6061 alloy cast rods. The chemical component of the alloy was determined by an optical emission spectrometer and shown in Table 1. The following paragraphs outline the specific test conditions:

Temperature: 370 °C, 420 °C, 470 °C, 520 °C

Strain: ε = 0.4, ε = 0.7, ε = 0.1, ε = 1.2

Strain rate: ε = 0.01 s^−1^, 0.1 s^−1^, 1.0 s^−1^, 5.0 s^−1^, 10.0 s^−1^

**Table 1:** The main alloying element content of the alloy (mass ratio/%).

Si	Mg	Fe	Cu	Mn	Cr	Zn	Ti	Al
0.474	0.853	0.1483	0.1747	0.0186	0.0565	0.0183	0.0142	Bal.

**Figure 4:**
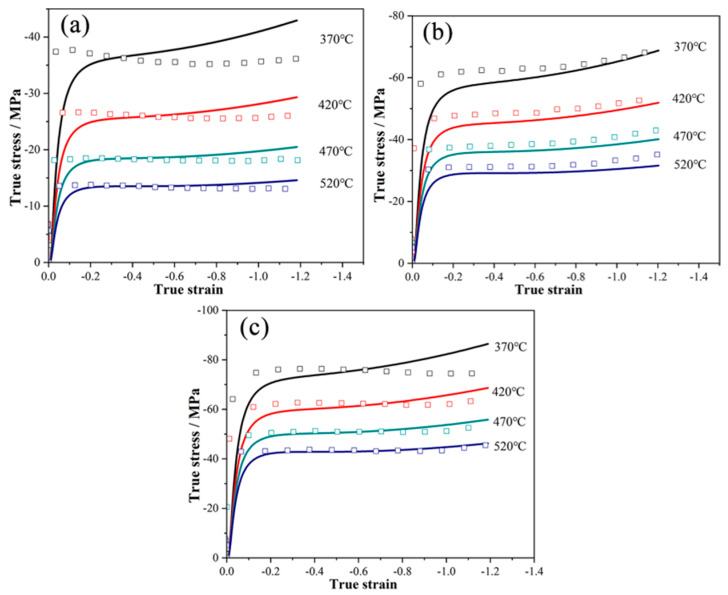
Comparison of predicted stress and experimental stress using the modified parameter model: (**a**) 0.01 s^−1^; (**b**) 1 s^−1^; (**c**) 10 s^−1^.

By conducting isothermal hot compression tests under different temperature and strain conditions and recording the corresponding mechanical behavior data, the stress–strain data of 6061 alloy under different deformation conditions can be obtained, as shown by the scattered hollow block symbols in Figure 4.

The default thermal deformation constitutive model of QFORM extrusion software is the Hansel–Spittel model, as shown in Formula (1). Based on the data obtained from isothermal hot compression tests, the thermal deformation constitutive equation can be fitted [20,21], and the actual parameters of the constitutive model under specific alloy composition conditions can be calculated, as shown in Table 2. The solid line curve in Figure 4 represents the predicted values of the thermal deformation constitutive equation.

(1) $$\sigma =A\cdot e^{m_{1}T}\cdot \varepsilon^{m_{2}}\cdot {\dot{\varepsilon }}^{m_{3}}\cdot e^{\frac{m_{4}}{\varepsilon }}\cdot {\left(1+\varepsilon \right)}^{m_{5}T}\cdot e^{m_{7}\varepsilon }\cdot {\dot{\varepsilon }}^{m_{8}\cdot T}\cdot T^{m_{9}}$$

where: σ—stress; ε—strain; ε̇—strain rate; T—temperature; A, m_1_~m_9_—relevant material parameters.

To evaluate the accuracy of the revised constitutive equation, the average relative error (AARE) was used to calculate all measured and predicted data [22]. Its expression is shown in Formula (2), where N is the total number of data used in this study and E_i_ and P_i_ are the experimental and predicted true stresses (MPa), respectively. Through calculation, the AARE value is 4.93%, indicating that the proposed constitutive model and calculated material constants can well describe the relationship between the rheological stress, temperature, strain rate, and strain of the studied material.

(2) $$AARE=\frac{1}{N}\sum_{i=1}^{N}\left|\frac{E_{i}-P_{i}}{E_{i}}\right|$$

### 2.2. Finite Element Simulation of Profile Forming

**Figure 5:**
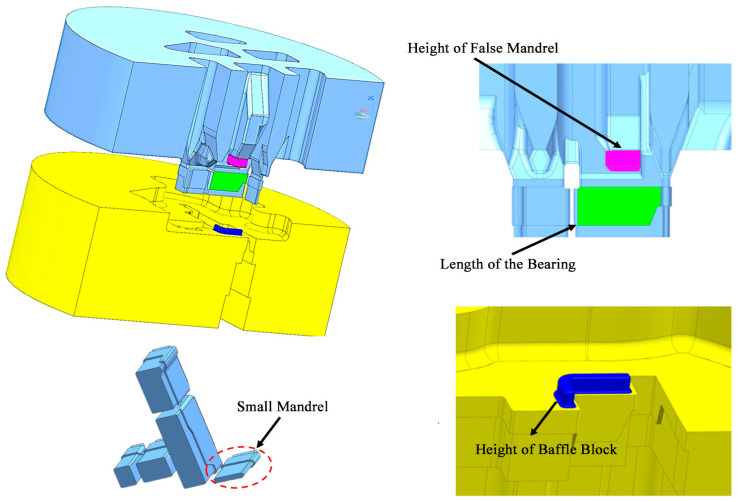
Three-dimensional structure of shunt die.

**Figure 6:**
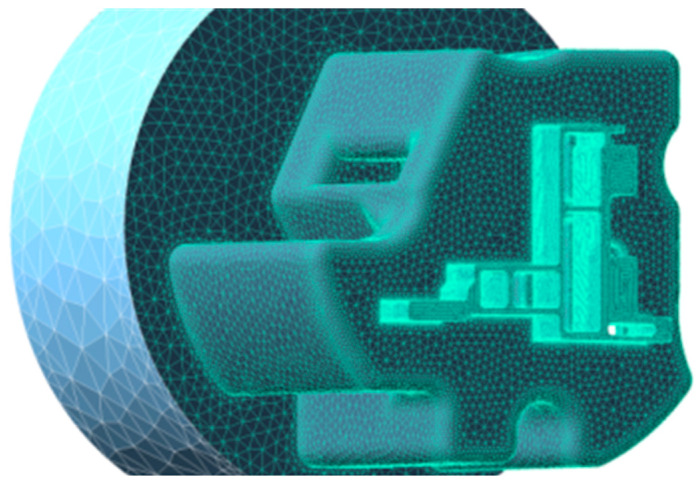
Mesh generation before 3D model simulation.

The profile shown in Figure 1 was selected as the research object, and the difficulty of die design for this product is shown in Figure 2. The area of thick wall A is 426 mm^2^, and the area of thin wall B is 42 mm^2^, with a tenfold difference. The stiffness of the small mandrel is insufficient. The deformation of the small mandrel caused by the superimposition of the aluminum flow velocity difference on both sides and the pressure difference affects the discharge flow velocity difference and pressure balance of the entire profile section. In the process of die design adjustment, structural change can easily cause quality abnormalities such as voids and looseness in area A. In this paper, the above design difficulties are characterized by three indicators: speed standard deviation (SDV), pressure standard deviation (SDP), and thick wall hydrostatic pressure (TWHP). The expressions of SDV and SDP are shown in Equations (3) and (4), where V_i_ is the velocity of node i along the extrusion direction on the cross-section and $\bar{V}$ is the average velocity of all nodes in the profile section, where n is the number of nodes [1,2,11,12]; similarly, P_i_ is the hydrostatic pressure at node i on the cross-section and $\bar{P}$ is the average static water pressure of all nodes in the cross-section of the profile, where n is the number of nodes, which can be directly read by QFROM extrusion. The TWHP is the average value of six points read from the QFORM within the geometric center of the area in Figure 2. The geometric model of the profile porthole extrusion die was established using SOLIDWORK 2016 software. The main structure of the die is shown in Figure 5 and Figure 6. The purple area in the figure represents the height of the false mandrel, the green area represents the length of the bearing, and the blue area represents the height of the baffle plates.

(3) $$\mathrm{SDV}=\sqrt{\frac{\sum_{i-1}^{n}{\left(V_{i}-\bar{V}\right)}^{2}}{n}}$$

(4) $$\mathrm{SDP}=\sqrt{\frac{\sum_{i-1}^{n}{\left(P_{i}-\bar{P}\right)}^{2}}{n}}$$

### 2.3. Box–Behnken Test Design

The Box–Behnken experiment is a commonly used design experiment method that is used to establish the relationship model between input variables (factors) and output response. It is a multi-factor and multi-level design method that can quickly and effectively determine the influence of factors on the response and optimize the experimental design. Each factor in the experimental design usually has three levels to capture the linear and quadratic effects of factors. Through the statistical analysis of the experimental results, the mathematical model between the response and factors can be established, and then the prediction, optimization, and parameter adjustment can be carried out [6,7,23].

In order to elucidate the functional relationship between the optimization objectives SDV, SDP, the maximum thick wall hydrostatic pressure, and the design variables, such as the height of baffle plates (0 mm, 3 mm, 6 mm), the length of the bearing (8 mm, 14 mm, 20 mm), and the height of the false mandrel (0 mm, 4 mm, 8 mm), the experimental design was carried out according to the Box–Behnken test method, and 17 three-dimensional geometric models of the die were constructed according to the test requirements.

### 2.4. Response Surface Method and NSGA2 Multi-Objective Optimization Genetic Algorithm

The response surface methodology (RSM) is an optimization method that combines the response surface from a set of experimental sample data, gives the surface equation, and then solves the surface equation to obtain a set of optimal design variables. Unlike other statistical methods, RSM not only considers the interaction between independent variables and improves the fitting accuracy but also utilizes graphical technology to display the functional relationship between the two, making the results more intuitive [6,7,23]. In this paper, the second-order response surface equation is selected, and its model can be expressed as follows:

(5) $$y=\beta_{0}+\sum_{i=1}^{n}\beta_{i}x_{i}+\sum_{i=1}^{n}\beta_{ii}x_{i}^{2}+\sum \sum_{P<i}\beta_{pi}x_{p}x_{i}+\varepsilon$$

**Figure 7:**
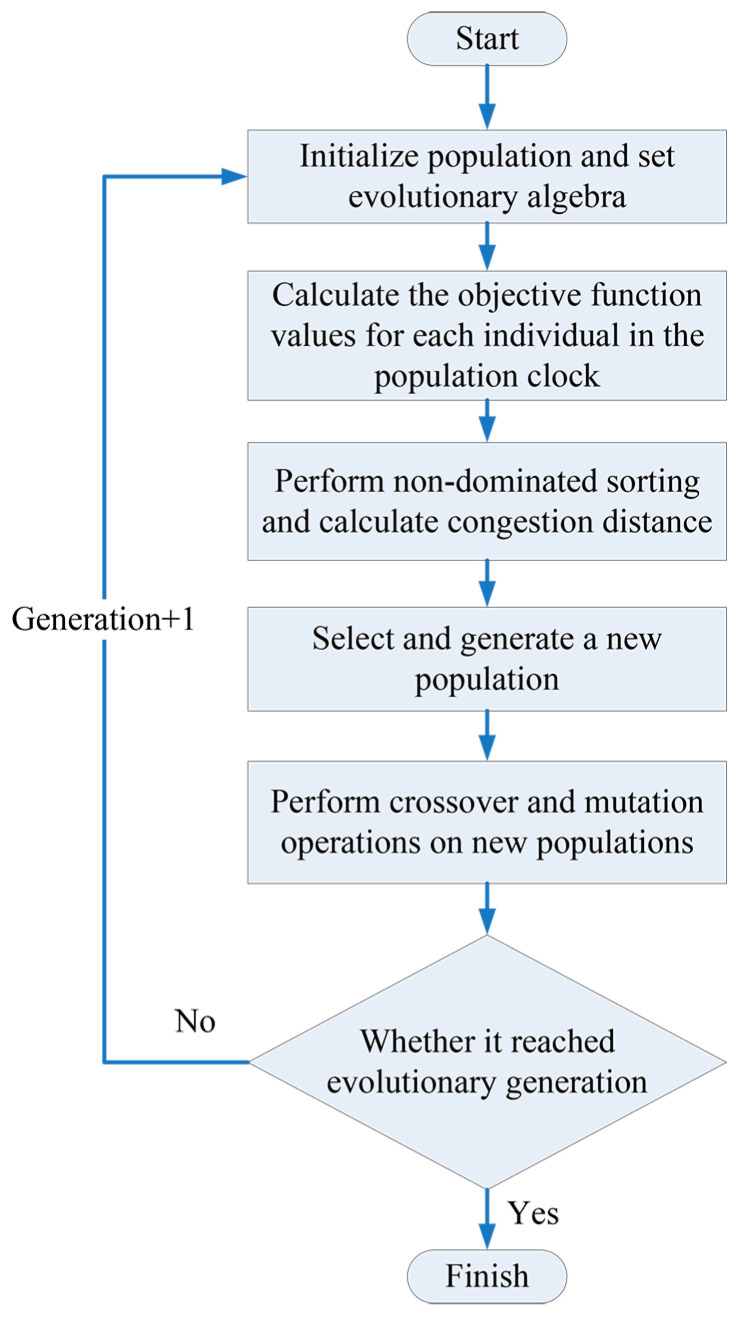
Multi-objective optimization flow chart of NSGA2 algorithm [28].

In recent years, various multi-objective optimization intelligent algorithms have been rapidly developed, and various algorithms with excellent performance indicators have emerged, such as DNEA, HREA, SMPSO, etc. Compared with these, the second-generation NSGA2 (non-dominated sorting genetic algorithm II) with an elite retention strategy does not have outstanding performance in fast non-dominated sorting algorithms [24]. However, as a classic multi-objective optimization algorithm, the NSGA2 algorithm has been successfully applied in multiple fields, proving its applicability and practicality. It has a solid theoretical and applied foundation. Thanks to the maturity of the algorithm, multiple open-source NSGA2 implementation tools are available, and researchers and engineers can easily apply the algorithm to their own problems. At the same time, some aspects of its performance, such as computational efficiency and convergence, still have certain advantages compared to other algorithms [25,26,27]. In this study, we use the NSGA2 algorithm to coordinate the calculation of the relationship between the three objective functions. The specific optimization process is shown in Figure 7.

### 2.5. Optimization Objectives and Multi-Objective Decision Making

The three objective function optimization objectives studied in this paper are shown in Formula (6):

(1) Min SDV(B,L,M) was designed to optimize the profile discharge balance.
(2) Min SDP(B,L,M)) was designed to stabilize the mandrel without deformation, and optimized the dimensional accuracy of the profile and the service life of the die.
(3) Max TWHP(B,L,M) was designed to ensure the internal structure of the profile was uniform and there were no fatal quality abnormalities such as porosity and voids.

(6) $$\left\{\begin{array}{c} \min\mathrm{SDV}(B,\;L,\;M)\\ \min\;\mathrm{SDP}(B,\;L,\;M)\\ \mathrm{maxTWHP}(B,\;L,M)\\ 0\leq B\leq 6\\ 8\leq L\leq 20\\ 0\leq M\leq 8 \end{array}\right.$$

where B is the height of the baffle plates and the value range is 0~6; L is the length of the bearing, with a value range of 8~20; M is the height of false mandrel, value range: 0~8.

It is usually impossible to obtain the optimal value of the three objective functions at the same time. How to choose or not needs to be judged by human subjectivity on the importance of each objective function. Based on the Pareto optimal solution set and subjective weight scoring, this paper uses TOPSIS method to evaluate and sort the items in the solution set to obtain the final solution [18]. The specific process is as follows:

(1) The subjective weights of the three indicators are calculated according to the expert scoring method (there are g experts in total):

(7) $$\beta_{n}=\sum_{n}^{g}A_{an}/g$$

where A_an_ is the scoring value of the nth index given by the expert.

(2) The Pareto optimal solution set has t solutions in total. Taking three objective functions as evaluation indexes, the index matrix s can be obtained.

S = (S_mn_)_t×3_ (m = 1,2,3,……t; n = 1,2,3)

(3) Normalize the matrix:

(8) $$S_{mn}^{*}=\left|\frac{S_{mn}-S_{max}}{S_{max}-S_{min}}\right|$$

(4) Weighting each element of the index matrix to obtain the weighting matrix K:

(9) $$K={\left(K_{mn}\right)}_{t\times 2}=\left(\beta_{1}\cdot S_{m1}^{*},\beta_{2}\cdot S_{m2}^{*},\beta_{3}\cdot S_{m3}^{*}\right)$$

(5) The minimum element of each column in the weighting matrix is taken as the optimal solution $Y_{n}^{+}$, and the maximum element in the weighting matrix is taken as the worst solution $Y_{n}^{-}$.

(10) $$Y_{n}^{+}=min(K_{1n,}K_{2n,}K_{3n}\cdots \cdots K_{tn})$$

(11) $$Y_{n}^{-}=max(K_{1n,}K_{2n,}K_{3n}\cdots \cdots K_{tn})$$

(6) Calculate the Euclidean distance (K_mn_) between each element in the weighting matrix and the optimal solution and the worst solution, $Z_{m}^{+}$, $Z_{m}^{-}$:
  (12) $$Z_{m}^{+}=\sqrt{\sum_{n=1}^{3}{\left(K_{mn}-Y_{n}^{+}\right)}^{2}}$$
  (13) $$Z_{m}^{-}=\sqrt{\sum_{n=1}^{3}{\left(K_{mn}-Y_{n}^{-}\right)}^{2}}$$

(7) The approximation index R_m_ between the m-th solution in the Pareto optimal solution set and the optimal level is calculated and sorted in descending order (the greater the R_m_, the closer it is to the optimal level):
  (14) $$R_{m}=\frac{Z_{m}^{-}}{Z_{m}^{-}+Z_{m}^{+}}$$

### 2.6. Hot Extrusion Production Verification

The hot extrusion production test uses a homogeneous 6061 round cast rod with a diameter of 228 mm and a length of 650 mm. The extruder is a 2500 t forward single-acting extruder from the profile factory affiliated to the Hesheng group. The aluminum rod is heated to 490 °C using a jet type fast heating gas furnace, and the extrusion die is placed in a resistance-type die heating furnace at 510 °C for 8 h. The preheating temperature of the extrusion barrel is 420 °C, and the propulsion speed of the master cylinder is 2.5 mm/s. After extrusion, the material head is reserved for analysis and die repair. After the residual material is cut off and the sandwich is shrunk, the middle part is taken for dimension measurement.

## 3. Test Analysis

### 3.1. Data Analysis and Establishment of Response Surface Function Relationship

In accordance with the Box–Behnken test design, finite element simulation analysis was carried out, and the relationship between 17 groups of design variables and the objective function was obtained, as shown in Table 3.

According to the data in Table 3, the stepwise regression method is used to model the response parameters in design expert 8.0, and the three quadratic regression models (the height of the material baffle plates (B), length of the bearing (L), and height of the false mandrel (M)) and three mass objectives (SDV, SDP, and thick wall hydrostatic pressure (TWHP)) are obtained, respectively. Table 4, Table 5 and Table 6 are variance analysis tables of each model.

It can be seen from Table 4 that the degree of freedom of the SDV regression model is 7, and the residual degree of freedom is 9. The F value of the regression equation was tested. By looking up the table of F-Vale under the conditions of different significance levels (α), it can be seen that F_0.05_(7, 9) = 3.29, F_0.025_(7, 9) = 4.20, and F_0.01_(7, 9) = 5.61. In Table 4, F = 56.82 is far greater than the F value under each significant level, indicating that the relationship between the SDV regression model and dependent variables is significant.

A P-test is conducted for each item in the regression equation. The item with *p* ≤ 0.05 has a significant impact on the dependent variable, the item with *p* ≤ 0.01 has a significant impact on the dependent variable, and the item with *p* > 0.5 has no significant impact on the dependent variable. Generally, this item is eliminated. After the BM and B^2^items are removed and recalculated, the *p* values in Table 4 are in line with the judgment of “extremely significant”.

“R-squared” is used to evaluate the fitting degree of the model, which is 0.9779, indicating that the model has strong explanatory ability. In order to prevent overfitting of the model, the “adj R-squared” value is introduced, which is 0.9607, further indicating the high goodness of fit of the mold [29]. According to the “pred R-squared” test, the goodness of fit between the predicted value of the model and the actual value is calculated to have a value of 0.8367, and the deviation from “adj R-squared” is small, indicating that the modified model has a better prediction ability. “Adeq precision” is used to measure the signal-to-noise ratio. Generally, a value greater than 4 proves that the model is desirable.

Based on the above analysis, the SDV regression model expression was finally obtained as follows:

SDV = +23.82148 − 0.77181×B − 0.86861×L − 0.67966×M + 0.031944×B×L + 0.021146×L×M + 0.021520×L^2^ + 0.021702×M^2^ (15)

The analysis steps in Table 4 were repeated to analyze Table 5 and Table 6, and the SDP 290 and thick wall hydrostatic pressure regression model expression were obtained as follows:

SDP = +15.26990 + 0.19583×B − 0.10021×L − 4.06250E − 003×M + 0.015000×B×M(16)

TWHP= +80.43597 − 3.64000×B − 3.98722×L − 3.07500×M − 0.13646×B×M + 0.41236×B^2^ + 0.15712×L^2^ + 0.13039×M^2^ (17)

In order to verify the prediction ability of RSM, in this paper, three additional groups of tests to use QFORM for numerical simulation were designed, and the results were compared with the prediction results of RSM. The results are shown in Table 7. The error between the predicted values and the simulation results is less than 7%, indicating that the prediction results of RSM are relatively accurate and highly consistent with the actual situation.

### 3.2. Response Surface Interaction Analysis

**Figure 8:**
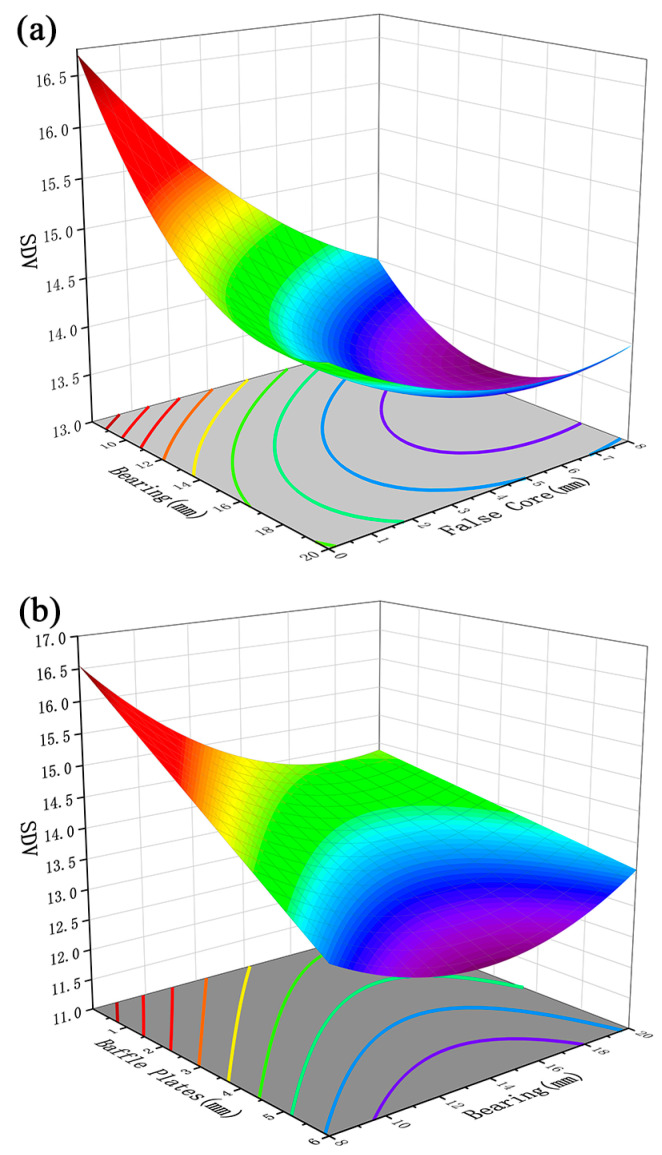
(**a**) Effect of bearing length and false mandrel height on SDV; (**b**) influence of baffle plate height and false mandrel height on SDV.

Figure 8a shows the effect of the interaction between the length of the bearing and the height of the false mandrel on the standard deviation of the section velocity. It can be observed from the figure that the standard deviation of the section velocity (SDV) decreases with the increase in the height of the false mandrel, indicating that the increase in the height of the false mandrel has a positive effect on the balance of the profile discharge. In addition, with the increase in the working band length, the SDV value first decreases and then increases, which indicates that there is a specific working band length to minimize the SDV value under this interaction condition. Figure 8b shows the effect of the interaction between the length of the bearing and the height of the baffle plates on the standard deviation of the section speed. Under this interactive condition, the SDV value will decrease with the increase in the height of the baffle plates, indicating that the greater the height of the baffle plates, the better the balance of the profile discharge. With the increase in the height of the bearing length, the SDV value first decreases and then increases, and the SDV value can be optimized under a certain bearing length.

The reason for the above phenomenon is that, in the process of profile forming, the resistance at the position of mass concentration on the section is small, resulting in a faster discharge speed at the thick wall compared to that at the thin wall. The greater the wall thickness difference, the greater the velocity difference. The adjustment of the flow rate can be conducted in two ways. The first way is to increase the resistance at the thick wall—that is, to increase the length of the bearing or the height of the baffle plates. The resistance is increased by increasing the friction force and changing the metal flow direction [19], and the resistance increases with the increase in the size of the two structures. However, it should be noted that if the resistance is too large, the metal flow at the thick wall will be excessively inhibited, resulting in an increase in the velocity difference on the profile section, which will lead to an increase in the standard deviation of the section velocity (SDV). The second way is to reduce the supply of metal at this position, which can be achieved by increasing the height of the false mandrel. Increasing the height of the false mandrel will reduce the volume of metal involved in forming, thus reducing the flow rate of metal. However, if the volume of metal involved in forming is too small, the corresponding section position of the false mandrel may not only cause the flow rate to be too slow but also may produce abnormal cavities.

As shown in Figure 8a,b, combined with the comparison of F values in Table 4, it can be seen that the order of primary and secondary factors affecting the SDV is B (144.55) > M (107.56) > L (40.24).

**Figure 9:**
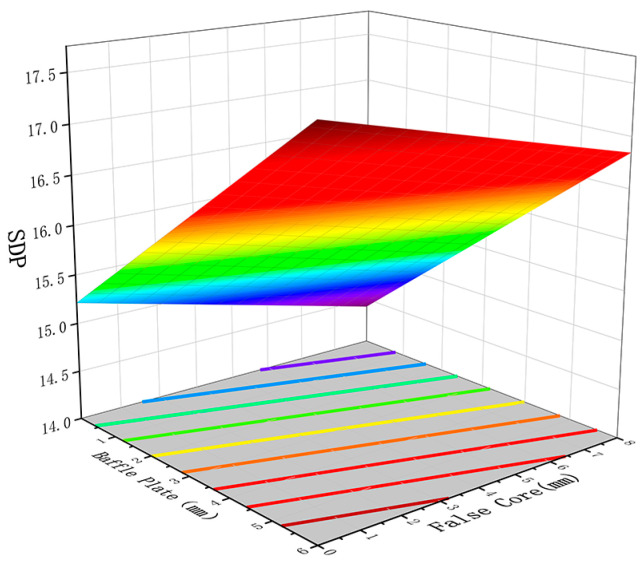
The influence of the height of the baffle plate and the height of the false core on the pressure standard deviation.

**Figure 10:**
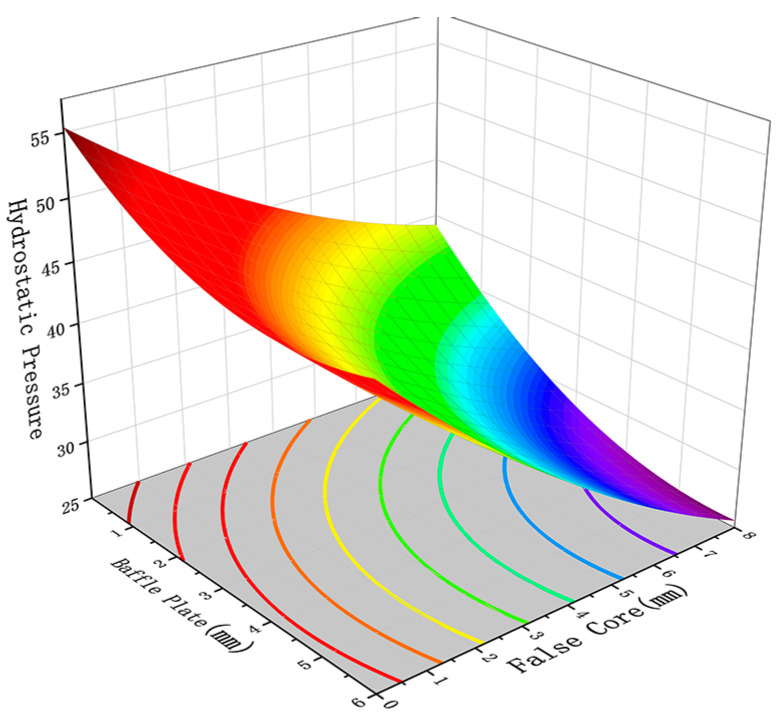
The influence of the height of the baffle plate and the height of the false core on the thick wall hydrostatic pressure.

Figure 9 and Figure 10 show the influence of the interaction between the height of the baffle plate and the height of the false mandrel on the pressure standard deviation and the pressure at the thick wall. As shown in Figure 9 and Figure 10, the increase in the height of the false mandrel and the height of the baffle plate will increase the standard deviation of the profile pressure and reduce the hydrostatic pressure at the thick wall. This is because the resistance of the thick wall part is small, resulting in the low distribution of hydrostatic pressure on the profile section, while the false core restricts the metal filling space, and the material blocking platform restricts the metal flow to a specific area. These two factors not only reduce the hydrostatic pressure at the thick wall but also increase the pressure instability of the entire profile section, resulting in the increase in the pressure standard deviation [30,31].

According to the comparison of F values in Table 5, the order of major and minor factors for the pressure standard deviation SDP is B (404.87) > L (248.47) > m (18.43). According to the comparison of F values in Table 6, the order of the primary and secondary factors for the TWHP is M (798.72) > B (220.87) > L (51.21).

### 3.3. NSGA2 Multi-Quality Objective Optimization

**Figure 11:**
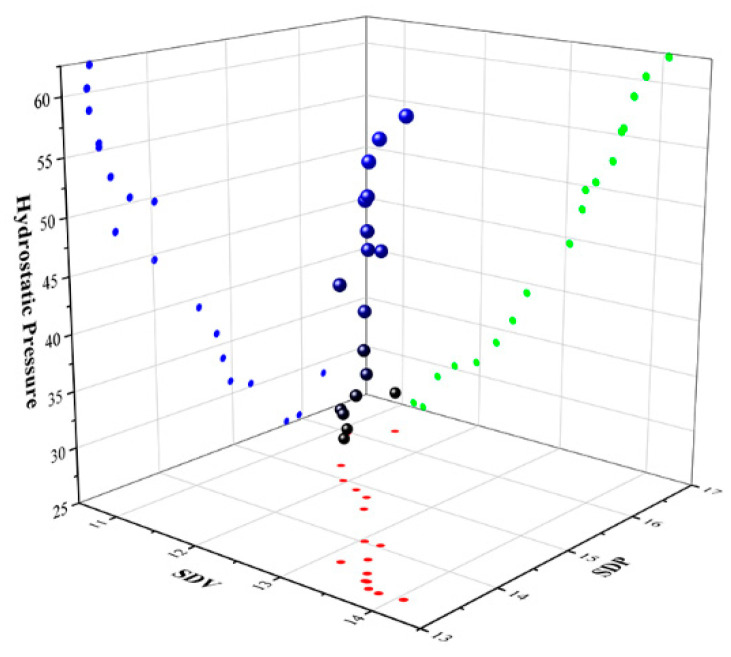
Pareto optimal solution set.

According to the analysis in Section 3.1 and Section 3.2, the three quality objectives examined in this paper (SDV, SDP, and TWHP) are independent and have certain internal relations. Finding a target to improve to the greatest extent without sacrificing the other two objectives constitutes a Pareto optimal solution set problem. This paper uses the NSGA2 multi-objective optimization genetic algorithm to solve the problem. The parameter settings are as follows: the initial population number is 100, the crossover probability is 0.8, the mutation probability is 0.1, and the number of iterations is 100. The Pareto optimal solution set is obtained through MATLAB 2022a programming, as shown in Figure 11.

### 3.4. Decision Results

Five experts scored the importance weight of the three optimization objectives. The scoring range of each objective was 0–1. The higher the score, the higher the importance. The total score of the three objectives was 1. The final score of each target was calculated using Formula (12), and the weight of the SDV and pressure standard deviation were determined. The weight of thick wall pressure was 0.3, 0.3, and 0.4, respectively. See Table 8 for specific scoring.

After obtaining the weight assignment, all 18 solutions in the Pareto optimal solution 380 set were sorted by the TOPSIS method, as shown in Table 9.

Taking the solution of No. 1 as the best scheme for the optimal design of the die in this paper, considering the actual design and manufacture, the optimal design parameters are confirmed to be as follows: a baffle block height of 0.0 mm, a bearing length of 20.0 mm, and a false mandrel height of 1.5 mm. A three-dimensional model was established according to the structural parameters of the optimal scheme and the initial scheme, and the extrusion process was simulated and compared using QFORM. The comparison of the simulation values of the two schemes is shown in Table 10. It can be seen from the table that the standard deviation of speed, pressure, and hydrostatic pressure of the thick wall of the optimal scheme were reduced by 5.33%, 11.10%, and 26.47%, respectively, compared with the initial scheme. The die designed and manufactured according to the optimal scheme successfully produced profiles and met the quality requirements. Thus, the effectiveness of the optimization of a complex multi-cavity aluminum extrusion die is proved.

**Figure 12:**
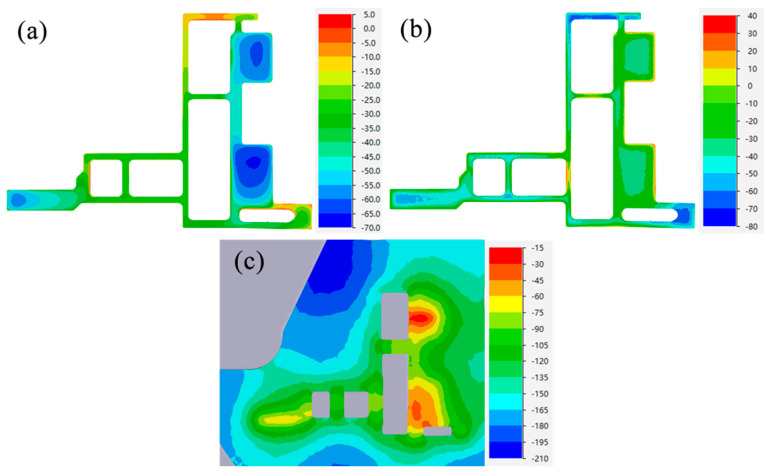
Initial design scheme: (**a**) section velocity distribution, (**b**) section hydrostatic pressure distribution, (**c**) thick wall hydrostatic pressure.

**Figure 13:**
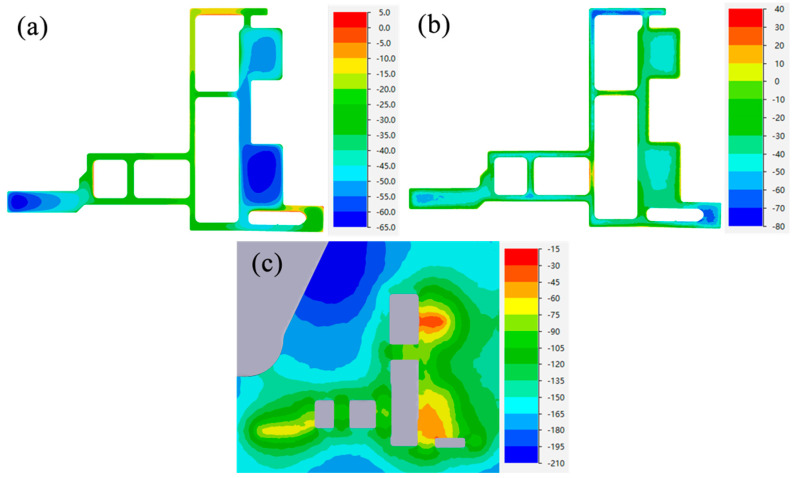
Optimization scheme: (**a**) section velocity distribution, (**b**) section hydrostatic pressure distribution, (**c**) thick wall hydrostatic pressure.

Figure 12 and Figure 13 show the profile section SDV, SDP and TWHP values at the die exit of different design schemes. From the comparison of Figure 12a and Figure 13a, it can be seen that there is little overall difference in the cross-section velocity distribution of the profiles in the two schemes, and the variation in the velocity difference is mainly concentrated on both sides of the small mandrel. The comparison between Figure 12b,c and Figure 13b,c shows that the pressure distribution changes are mainly concentrated in thick wall area A, the hydrostatic pressure value of the optimized thick wall area A is significantly increased, and the distribution range of a high pressure value is increased. It can be seen that when the length of the bearing increases from the initial design of 8 mm to 20 mm, the blocking effect caused by friction restricts the flow of metal in thick wall area A, while the height of the false mandrel decreases from 4 mm to 1.5 mm and the baffle plate is completely removed, promoting more metal to gather in the thick wall area and increasing the volume of metal actually participating in the formation. Under the combined action of the two, the relative velocity in area A decreases and the hydrostatic pressure increases, which plays a role in balancing the velocity and pressure on the profile section.

**Figure 14:**
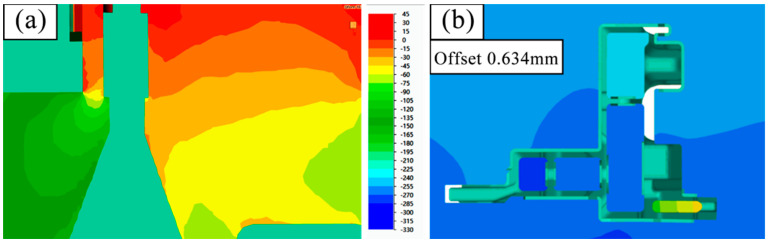
(**a**) Pressure difference on both sides of core C and (**b**) Y-direction deflection in the initial design scheme.

**Figure 15:**
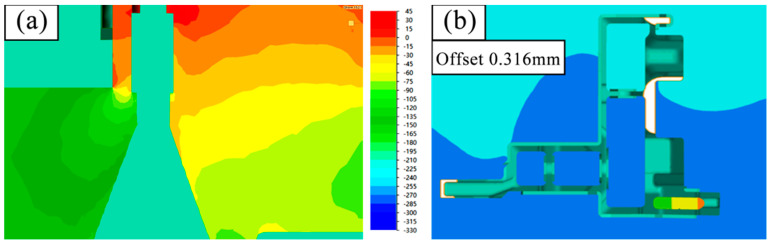
(**a**) Optimal design scheme hydrostatic pressure on both sides of core C and (**b**) Y-direction deflection.

Figure 14 and Figure 15 show the initial design scheme and the optimized design scheme, showcasing the pressure difference on both sides of the mandrel and the deflection of the mandrel. As shown in the figures, 10 symmetrical points are taken on both sides of the mandrel, respectively, and the arithmetic mean after reading the average stress value is calculated. In Figure 14, the average stress value near area A is 38.42 MPa and the average stress value near area B is 121.82 MPa; in Figure 15, the average stress value near area A is 45.27 MPa and the average stress value near area B is 119.94 MPa. Through comparison, it can be seen that design optimization results in an approximately 18% increase in the average stress at the thick wall side of the mandrel under the condition that the pressure at the thin wall side of the small mandrel is basically unchanged. The effect of this on the mandrel is that the deflection deformation in the Y direction is reduced from 0.634 mm under the initial design conditions to 0.316 mm.

### 3.5. Verification of Hot Extrusion Test

**Figure 16:**
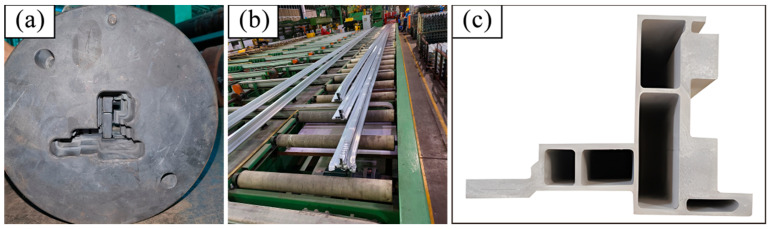
(**a**) Physical drawing of die; (**b**) extrusion production process; (**c**) macrostructure of profile.

Finally, the optimal design parameters of the die were determined to be as follows: a baffle block height of 0 mm, a bearing length of 20 mm, and a false mandrel height of 1.5 mm. The die was designed and manufactured accordingly (as show in Figure 16a) and the hot extrusion trial production was carried out according to the process conditions determined in Section 1 (as shown in Figure 16b). After the trial production was completed, the dimensions of the profile were inspected according to the product design drawings. As outlined in Table 11, the product dimensional tolerance met the design requirements, and the profile dimensional tolerance requirements could be met after slight local adjustment of the die repair. Figure 16c shows the macroscopic metallographic observation results of the profile, from which no voids or loose abnormal structures were found. The structural density must meet the specified design criteria.

## 4. Conclusions

(1) This study achieved significant innovative results in the design of multi-cavity extrusion profiles with significant wall thickness differences for new energy vehicles. By analyzing the specific requirements of the new profile structure in detail, this work introduced two new evaluation indicators: the velocity standard deviation (SDV) and thick wall hydrostatic pressure (TWHP). These two indicators not only provide new dimensions for quality evaluation in the extrusion process of profiles but also verify their significant correlation with quality objectives through analysis of variance, proving their effectiveness in optimizing die design and improving profile quality.
(2) In addition, the FEM analysis software and response surface analysis method used in this study established an accurate mathematical model for the complex relationship between die design parameters and profile quality. Using the NSGA2 multi-objective genetic optimization algorithm, this work optimized the velocity standard deviation (SDV), pressure standard deviation (SDP), and thick wall hydrostatic pressure (TWHP). The research results show that compared with the initial design scheme, the optimized die design reduces the standard deviation of profile section velocity by 5.33%, the standard deviation of pressure by 11.16%, and the static water pressure in the thick wall area by 26.47%. These improvements demonstrate the effectiveness of this study in optimizing the design of complex aluminum profile dies with significant wall thickness differences in multiple cavities.
(3) The optimized die was successfully applied in actual manufacturing and achieved success in subsequent extrusion production experiments, effectively ensuring the dimensional accuracy and metallographic structure quality of the profile. This achievement not only improves the production efficiency and quality of profiles but also provides a new solution for multi-objective optimization of extrusion die design, and it also provides valuable experience and reference for research and practice in related fields.

## Figures and Tables

**Table 2:** Modification of Hansel–Spittel flow stress model.

Coefficient	m_1_	m_2_	m_3_	m_4_	m_5_	m_7_	m_8_	m_9_	A
Value	−4.82 × 10^−4^	−0.167	−0.062	−0.044	−1.21 × 10^−3^	0.59	4.390 × 10^−4^	−1.65	1.065 × 10^−6^

**Table 3:** Box–Behnken test design parameters and results.

Number	B/mm	L/mm	M/mm	SDV	SDP	TWHP (MPa)
1	3	14	4	13.72	14.58	36.35
2	3	20	0	14.69	13.79	57.76
3	6	8	4	13.27	15.94	39.7
4	6	14	0	13.68	15	48.18
5	0	8	4	16.15	14.45	48.4
6	3	14	4	13.72	14.58	36.35
7	6	14	8	12.58	15.81	25.3
8	6	20	4	13.39	14.87	42.01
9	3	8	8	13.98	15.37	31.75
10	3	8	0	17.02	15.26	49.88
11	3	20	8	13.68	14.09	36.98
12	0	20	4	14.56	13.46	52.76
13	0	14	0	15.78	13.74	55.72
14	3	14	4	13.72	14.58	36.35
15	3	14	4	13.72	14.58	36.35
16	0	14	8	14.21	13.83	39.39
17	3	14	4	13.72	14.58	36.35

**Table 4:** Regression model analysis of speed standard deviation.

Variance Source	Sum of Squares	Degree of Freedom	Mean Variance	F Value	*p* Value
Regression model	20.87	7	2.98	56.82	<0.0001
B	7.59	1	7.59	144.55	<0.0001
L	2.11	1	2.11	40.24	0.0001
M	5.64	1	5.64	107.56	<0.0001
BL	1.32	1	1.32	25.2	0.0007
LM	1.03	1	1.03	19.63	0.0016
L^2^	2.53	1	2.53	48.29	<0.0001
M^2^	0.51	1	0.51	9.7	0.0124
Residual	0.47	9	0.052	—	—
Misfit	0.47	5	0.094	—	—
Pure error	0	4	0	—	—
Total	21.35	16	—	—	—
R-Squared = 0.9779, Adj R-Squared = 0.9607. S-Pred R-Squared = 0.8367, Adeq Precision = 28.315.					

**Table 5:** Regression model analysis of pressure standard deviation.

Variance Source	Sum of Squares	Degree of Freedom	Mean Variance	F Value	*p* Value
Regression model	7.95	4	1.99	170.73	<0.0001
B	4.71	1	4.71	404.87	<0.0001
L	2.89	1	2.89	248.47	<0.0001
M	0.21	1	0.21	18.43	0.001
BM	0.13	1	0.13	11.13	0.0059
Residual	0.14	12	0.012	—	—
Misfit	0.14	8	0.017	—	—
Pure error	0	4	0	—	—
Total	8.09	16	—	—	—
R-Squared = 0.9827, Adj R-Squared = 0.9770. S-Pred R-Squared = 0.9555, Adeq Precision = 46.787					

**Table 6:** Regression model analysis of thick wall hydrostatic pressure.

Variance Source	Sum of Squares	Degree of Freedom	Mean Variance	F Value	*p* Value
Regression model	1264.76	7	180.68	189.18	<0.0001
B	210.95	1	210.95	220.87	<0.0001
L	48.91	1	48.91	51.21	<0.0001
M	762.84	1	762.84	798.72	<0.0001
BM	10.73	1	10.73	11.23	0.0085
B^2^	57.99	1	57.99	60.72	<0.0001
L^2^	134.71	1	134.71	141.04	<0.0001
M^2^	18.33	1	18.33	19.19	0.0018
Residual	8.6	9	0.96	—	—
Misfit	8.6	5	1.72	—	—
Pure error	0	4	0	—	—
Total	1273.36	16	—	—	—
R-Squared = 0.9932, Adj R-Squared = 0.9880. S-Pred R-Squared = 0.9701, Adeq Precision = 45.823					

**Table 7:** Comparison between RSM prediction results and numerical analysis results.

Number	B/mm	L/mm	M/mm	Analog Value			RSM Predicted Value			Error		
				SDV	SDP	TWHP	SDV	SDP	TWHP	SDV	SDP	TWHP
1	0	20	1	14.63	13.38	57.97	14.82	13.26	60.59	1.30%	0.90%	4.52%
2	3.5	19	2	14.13	13.91	50.34	14.07	14.10	47.35	0.40%	1.39%	6.32%
3	6	12	8	13	16.08	25.13	12.15	15.93	25.41	6.54%	0.93%	1.11%

Remarks: B is the height of the baffle plates, L is the length of the bearing, and M is the height pof the false mandrel.

**Table 8:** Expert scoring.

Expert Serial Number	Index Weight of SDV	Index Weight of SDP	Weight of TWHP
1	0.2	0.3	0.5
2	0.3	0.3	0.4
3	0.2	0.4	0.4
4	0.3	0.2	0.5
5	0.3	0.4	0.3
Arithmetic mean	0.3	0.3	0.4

**Table 9:** Order of Pareto optimal solution set obtained by decision.

Serial Number	Design Variable			Target			Index Close to Optimal Level Rm
	Baffle Block	Bearing	False Mandrel	SDV	SDP	TWHP	
1	0.11	19.82	1.47	13.71	13.30	58.50	0.64
2	0.07	19.90	0.92	13.83	13.29	60.34	0.64
3	0.03	19.47	0.01	14.06	13.32	62.23	0.62
4	0.27	19.19	1.81	13.61	13.40	55.61	0.62
5	0.27	19.19	1.94	13.59	13.40	55.28	0.62
6	0.53	18.56	2.14	13.50	13.52	52.60	0.60
7	1.84	19.33	2.06	13.32	13.74	50.47	0.59
8	0.79	18.95	4.11	13.18	13.56	47.81	0.59
9	3.29	19.37	1.51	13.21	14.04	49.61	0.57
10	2.22	17.62	2.79	13.05	14.02	44.49	0.53
11	3.88	16.24	3.18	12.56	14.57	39.07	0.45
12	4.08	14.78	3.72	12.40	14.80	36.14	0.40
13	4.15	14.71	4.66	12.20	14.88	33.63	0.39
14	4.54	15.52	5.86	11.96	14.98	31.20	0.38
15	5.45	15.54	6.14	11.68	15.26	30.30	0.37
16	6.00	11.75	8.00	11.15	15.95	25.49	0.36
17	5.70	12.70	8.00	11.27	15.76	25.32	0.35
18	5.99	8.02	7.97	11.46	16.32	28.81	0.33

**Table 10:** Comparison results between the best scheme and the initial scheme.

Scheme Category	Design Variable			Analog Value		
	B/mm	L/mm	M/mm	SDV	SDP	TWHP
Initial plan	2	8	4	15.38	15.05	43.87
Optimal scheme	0	20	1.5	14.56	13.37	55.481
Optimization and promotion	/	/	/	5.33%	11.16%	26.47%

**Table 11:** Partial dimension test results.

Drawing	Measured	Determine
122.02 ± 0.35	122.08	OK
23.5 ± 0.2	23.36	OK
56 ± 0.25	55.92	OK
30 ± 0.2	29.86	OK
8 ± 0.2	8.07–8.03	OK
2.5 + 0.4/−0.2	2.67	OK
17.5 + 0/−0.3	17.46–17.42	OK
16.5 + 0.4/0	16.62–16.54	OK
20 + 0.4/0	20.12–20.08	OK
16.5 + 0.4/0	16.51–16.48	NG
23.32 + 0.4/0	23.42	OK
10 ± 0.2	10.02–8.86	OK

## Data Availability

Data are contained within the article.
